# A dataset composed of multiangular spectral libraries and auxiliary data at tree, leaf, needle, and bark level for three common European tree species

**DOI:** 10.1016/j.dib.2021.106820

**Published:** 2021-01-30

**Authors:** Aarne Hovi, Petri R. Forsström, Giulia Ghielmetti, Michael E. Schaepman, Miina Rautiainen

**Affiliations:** a Department of Built Environment, Aalto University, School of Engineering, P.O. Box 14100, FI-00076 Aalto, Finland; b Department of Geography, Remote Sensing Laboratories, University of Zürich, Winterthurerstrasse 190, CH-8057 Zurich, Switzerland; c Department of Electronics and Nanoengineering, Aalto University, School of Electrical Engineering, P.O. Box 15500, FI-00076 Aalto, Finland

**Keywords:** Forest, Tree, Multiangular, Satellite, Goniometer, BRDF, Reflectance, Transmittance

## Abstract

This article describes a dataset of multiangular scattering properties of small trees (height = 0.38–0.7 m) at visible, near-infrared, and shortwave-infrared wavelengths (350–2500 nm), and provides supporting auxiliary data that comprise leaf, needle, and bark spectra, and structural characteristics of the trees. Multiangular spectra were measured for 18 trees belonging to three common European tree species: Scots pine (*Pinus sylvestris* L.), Norway spruce (*Picea abies* (L.) H. Karst), and sessile oak (*Quercus petraea* (Matt.) Liebl.). The measurements were performed in 47 different view angles across a hemisphere, using a laboratory goniometer and a non-imaging spectrometer. Leaf and needle spectra were measured for each tree, using a non-imaging spectrometer coupled to an integrating sphere. Bark spectra were measured for one sample tree per species. In addition, leaf and needle fresh mass, surface area of leaves, needles, and woody parts, silhouette area, and spherically averaged silhouette to total area ratio (STAR) for each tree were measured or derived from the measurements. The data are useful for modeling the shortwave reflectance characteristics of small trees and potentially forests, and thus benefit climate modeling or interpretation of remote sensing data.

## Specifications Table

**Table:** 

Subject	Environmental engineering
Specific subject area	Remote sensing of forests; radiative transfer modeling; land surface modeling
Type of data	Table Image
How data were acquired	Five different types of measurements were conducted for each of the 18 trees, according to the following protocol: 1. Multiangular spectra of individual trees: Shortwave radiation scattered by the tree, illuminated with a halogen lamp from a zenith angle of 40°, were measured in 47 different view angles across a hemisphere, using a biconical measurement setup. A goniometer was used in combination with an ASD FieldSpec 3 non-imaging spectrometer, operating at wavelengths 350–2500 nm and producing spectra at 1 nm interval. 2. Multiangular silhouette area of individual trees: Digital photographs of the tree were acquired against a white background using the same 47 view angles as described above, and additionally in the direction of the illumination. The photographs were processed to multiangular silhouette area by thresholding the blue channel to produce a black-and-white image. 3. Leaf and needle spectra: Three samples of leaves or needles were picked from the tree, and leaf and needle directional-hemispherical reflectance and transmittance spectra were measured using an ASD FieldSpec 3 non-imaging spectrometer attached to an ASD RTS-3ZC integrating sphere with a halogen light source. 4. Leaf and needle mass and area: All leaves or needles were picked from the tree and weighed with a precision scale. A subset of leaves or needles was measured for the projected area (Epson Perfection V550 scanner) and another subset (conifers only) was measured for needle dimensions (manual measurement with a digital calliper). Leaf or needle projected area to fresh mass and total surface area to projected area ratios were determined, and total leaf or needle surface area was calculated. 5. Woody area: Finally, the tree without foliage was photographed within the goniometer to determine multiangular silhouette areas of the woody parts. The photographs were further processed to total surface area of the woody parts. Additionally, directional-hemispherical reflectance spectra of bark samples from three selected trees (one per species) were measured, applying the same measurement setup as used for the leaf and needle spectra in step 3 above.
Data format	Raw Filtered
Parameters for data collection	Data were collected during late growing season (from Aug 20th to Sep 20th) in 2018, so that the leaves in the trees were still fully green. All measured trees were living and healthy, and were stored in an outdoor garden and watered frequently during the campaign. All measurements were conducted in a laboratory, i.e., indoors and in stable temperature. One to two trees per day were measured. A tree was brought into the laboratory just before starting the measurements. Multiangular spectral measurements were performed within 1–1.5 h, and leaf and needle spectral measurements within approximately 6 h from the start of the measurements.
Description of data collection	The trees were selected from a pool of trees grown outdoors in a tree nursery. The selection process maximized variation in crown structure (spherically averaged silhouette to total area ratio). Crown structure was hypothesized to affect scattering properties of the trees. Most of the trees were transported from the tree nursery (located in Zurich) to the garden of University of Zurich (located at walking distance from the measurement laboratory) at the start of the campaign. Few additional trees were transported from the nursery in the middle of the campaign.
Data source location	Zurich Switzerland 47°23′N, 8°33′E
Data accessibility	Repository name: Mendeley data Data identification number: 10.17632/7myhzwz6w9.1 Direct URL to data: http://dx.doi.org/10.17632/7myhzwz6w9.1
Related research article	P.R. Forsström, A. Hovi, G. Ghielmetti, M.E. Schaepman, M. Rautiainen, Multi-angular reflectance spectra of small single trees. Remote Sens. Environ. 255 (2021) 112302. https://doi.org/10.1016/j.rse.2021.112302

## Value of the Data

• The dataset includes spectral libraries of trees, leaves, needles, and bark for three common tree species in Europe, and has thus versatile uses in remote sensing, ecosystem and climate modeling.
• These data can benefit 1) developers of (radiative transfer) models characterizing the shortwave radiation regime of vegetation, 2) developers of remote sensing methods and applications aiming at detecting biodiversity metrics or individual species, and 3) developers of land surface (climate) models.
• These data might be further used, for example, 1) for designing new laboratory measurement set-ups for spectral properties of vegetation or other non-flat (volumetric) targets, 2) for validating radiative transfer models of single trees, or 3) as reference spectral libraries as needed in analyses of optical remote sensing data from forest-covered landscapes.

## 1 Data Description

The dataset comprises mainly tabular data, and is organized in five groups (Table 1). Group 1 contains one table (‘*trees.csv*’) that lists the unique tree ID numbers, metadata such as dates of measurement, and structural parameters of the trees. Group 2 contains two tables for storing the phytoelement (leaf, needle, and bark) spectra: ‘*elementspectra-list_of_samples.csv*’ lists the measured samples and their metadata, and ‘*elementspectra-spectra.csv*’ contains the spectra. Group 3 contains data from the multiangular spectral measurements of trees in the goniometer, and Group 4 contains the data from the silhouette photography of the trees. The most important files in Groups 3–4 are ‘*treespectra-angles.csv*’, which lists the measured trees and view angles, and ‘*treespectra-DSC_tree_raw.csv*’ and ‘*treespectra-DSC_tree_filt.csv*’ which contain the directional scattering coefficients (DSC, [sr^−1^]) of the trees in all view angles. The first file contains unfiltered (used in Forsström et al. [1]) and the second contains filtered spectra (used in Hovi et al. [2]). The other files in Groups 3–4 contain raw measurement data or parameters that are used for calculating the DSCs or structural parameters of the trees. These files are required only if one wants to repeat or modify the data processing. The naming of the files follows the symbols used in our equations (see Section 2). A Python code for reading and visualizing the data (‘*read_and_visualize_data.py*’) with some examples of data processing is provided together with the data. The measurement theory, equations, and processing steps are also described in Section 2 of this article. Finally, Group 5 comprises auxiliary files that are used in the data interpretation or processing, including wavelengths of the spectral data (‘*aux-wavelengths-csv*’), reflectance spectrum of the white reference panel used in the goniometer measurements (‘*aux-R_WR_tree.csv*’), and reflectance spectrum of the background canvas used in the measurements (‘*aux-R_canvas.csv*’). All data are in comma-separated .csv format with UTF-8 encoding, except photographs of the tree silhouettes, which are in .png format (Table 1). In the files that contain spectra, there are always 2151 columns, named as ‘wl350’ to ‘wl2500’, denoting wavelengths of 350–2500 nm. The contents of the other files are explained in Table 2, Table 3, Table 4, Table 5, Table 6.

**Table 1:** Summary of data files included in the dataset.

File name	Description	Rows	Columns
**Group 1: Trees, their metadata, and structure**			
*trees.csv*	List of measured trees, metadata, and structural parameters	19^1^	17^2^
**Group 2: Leaf, needle and bark spectra**			
*elementspectra-list_of_samples.csv*	List of measured leaf, needle, and bark samples and their metadata	227^4^	4^2^
*elementspectra-spectra.csv*	Table containing the measured spectra [DHRF, DHTF]^3^	227	2151
**Group 3: Multiangular spectra of trees measured in the goniometer**			
*treespectra-angles.csv*	List of tree IDs and measured azimuth and zenith angles	972^5^	3^2^
*treespectra-DSC_tree_raw.csv*	Unfiltered directional scattering coefficients of the trees, used in Forsström et al. [1] [sr^−1^]	972	2151
*treespectra-DSC_tree_filt.csv*	Filtered and jump-corrected directional scattering coefficients of the trees, used in Hovi et al. [2] [sr^−1^]	972	2151
*treespectra-DN_total_tree.csv*	Raw (unprocessed) digital numbers recorded for the trees by the spectrometer [digital numbers]	972	2151
*treespectra-DN_stray_tree.csv*	Estimated stray light signal for each tree and view angle [digital numbers]	972	2151
*treespectra-b_tree.csv*	Fraction of stray light not obscured by the tree [fraction]	972	3^2^
*treespectra-f_tree.csv*	Signal recorded from the tree compared to a signal recorded by an isotropic detector [fraction]	972	3^2^
*treespectra-DN_total_WR_tree.csv*	Raw (unprocessed) digital numbers recorded for the white reference by the spectrometer [digital numbers]	18^6^	2151
*treespectra-DN_stray_WR_tree.csv*	Stray light recorded for the white reference [digital numbers]	18	2151
*treespectra-b_WR_tree.csv*	Fraction of stray light not obscured by the white reference panel [fraction]	18	3^2^
*treespectra-f_WR_tree.csv*	Signal recorded from the white reference compared to a signal recorded by an isotropic detector [fraction]	18	3^2^
**Group 4: Multiangular silhouette photography of trees performed in the goniometer**			
*silhouettes-S_tree.csv*	Silhouette areas of the tree in each view angle, and in the direction of illumination [m^2^]	990^7^	4^2^
*silhouettes-S_tree_wood.csv*	Silhouette area of the tree without foliage, in selected view angles [m^2^]	378^8^	4^2^
*silhouettes-photographs.zip*	Silhouette photographs in .png format; file names indicate tree ID and azimuth/zenith angles^9^	–	–
*silhouettes-photographs_wood.zip*	Silhouette photographs without foliage in .png format; file names indicate tree ID and azimuth/zenith angles^9^	–	–
**Group 5: Auxiliary files**			
*aux-wavelengths.csv*	List of wavelengths recorded by the spectrometer [nm]	1	2151
*aux-R_WR_tree.csv*	Reflectance spectrum of the white reference panel used in the goniometer measurements [DHRF]^3^	1	2151
*aux-R_canvas.csv*	Reflectance spectrum of the background canvas used in the goniometer measurements [DHRF]^3^	1	2151

1 There are 18 trees for which all measurements except bark spectra were performed. Bark spectra were measured for three trees, including one oak tree outside of the group of 18.

2 See Table 2, Table 3, Table 4, Table 5, Table 6 for explanation of the headers of these files.

3 Directional-hemispherical reflectance- and transmittance factors.

4 There are 216 leaf or needle, and 11 bark spectra. See Section 2.2.3 for description of the sampling for each tree.

5 There are 18 trees, and 54 view angles (of which 47 unique, and 7 repetitions in nadir) for each tree.

6 There are 18 trees, and one measurement for each tree.

7 There are 18 trees, and 54 view angles (of which 47 unique, and 7 repetitions in nadir) for each tree. In addition, one illumination angle for each tree.

8 There are 18 trees, and 21 view angles (of which 19 unique, and 2 repetitions in nadir) per tree.

9 All images have been masked with a polygon that delineates the area that contains the tree and white background. Other processing has not been applied, i.e., the images are as outputted by the camera.

**Table 2:** Header of ‘*trees.csv*’.

Variable	Explanation
tree_ID	Unique tree ID
species	Tree species
bark_measured	Were bark spectra measured? (1 = yes, 0 = no)
other_measured	Were all other measurements performed? (1 = yes, 0 = no)
date	Measurement date
canvas_height	Height of the background canvas from the floor [cm]
tree_height	Height of the tree [cm]
FM	Fresh mass of all foliage in the tree [g]
PA_to_FM	Projected area to fresh mass ratio for foliage [m^2^ kg^−1^]
TA_to_PA	Total (surface) area to projected area ratio for foliage [m^2^ m^−2^]
TA_foliage	Total (surface) area of foliage in the tree [m^2^]
TA_wood	Total (surface) area of woody parts in the tree [m^2^]
TA_all	Total (surface) area of all phytoelements (foliage + woody parts) in the tree [m^2^]
sph_avg_S_tree_all	Spherically averaged silhouette area of the tree [m^2^]
sph_avg_S_tree_wood	Spherically averaged silhouette area of the tree without foliage [m^2^]
STAR_foliage	Spherically averaged sihouette to total area ratio, calculated by counting only foliage to the total area (i.e., STAR_foliage = sph_avg_S_all / TA_foliage) [fraction]
STAR_all	Spherically averaged sihouette to total area ratio, calculated by counting all phytoelements (foliage + woody parts) to the total area (i.e., STAR_all = sph_avg_S_all / TA_all) [fraction]

**Table 3:** Header of ‘*elementspectra-list_of_samples.csv*’.

Variable	Explanation
tree_ID	Unique tree ID
element	Name of the element: foliage or wood
sample_nr	Number of the sample (1 to 3) in a tree
side	Side of leaf: A = adaxial i.e. ‘upper’ side, B = abaxial i.e. ‘lower’ side. Note that spruce needles were symmetric and no adaxial and abaxial sides could be distinguished. Both sides of the sample were measured also for spruce needles, but ‘A’ and ‘B’ have no meaning.
quantity	Quantity that was measured: R = reflectance [HDRF], T = transmittance [HDTF]

**Table 4:** Header of ‘*treespectra-angles.csv*’. For angle notation, e.g., directions of negative and positive zenith angles in each azimuth, see Fig. 2.

Variable	Explanation
tree_ID	Unique tree ID
azimuth	View azimuth angle [°]
zenith	View zenith angle [°]

**Table 5:** Header of ‘*treespectra-b_tree.csv*‘, ‘*treespectra-f_tree.csv*’, ‘*refspectra-b_WR_tree.csv*‘, and ‘*refspectra-f_WR_tree.csv*‘.

Variable	Explanation
VNIR	Value of *f* or *b* for the VNIR detector (350–1000 nm) [fraction]
SWIR1	Value of *f* or *b* for the SWIR1 detector (1001–1800 nm) [fraction]
SWIR2	Value of *f* or *b* for the SWIR2 detector (1801–2500 nm) [fraction]

**Table 6:** Header of ‘*silhouettes-S_tree.csv*‘, and ‘*silhouettes-S_tree_wood.csv*‘. For angle notation, e.g., directions of negative and positive zenith angles in each azimuth, see Fig. 2.

Variable	Explanation
tree_ID	Unique tree ID
azimuth	Azimuth angle [°]
zenith	Zenith angle [°]
silhouette_area	Silhouette area of the tree [m^2^]

## 2 Experimental Design, Materials and Methods

### 2.1 Overview of the experiment and measured trees

The experiment was conducted in a goniometer laboratory located at the University of Zurich, Remote Sensing Laboratories. The data comprise measurements of 18 small trees of 0.38–0.7 m in height and up to 4 years in age. The measurement routine for one tree was as follows: i) The tree was brought inside to the laboratory and its multiangular spectra were measured in the goniometer, ii) the tree was photographed in the goniometer to obtain multiangular silhouette areas, iii) reflectance and transmittance spectra of the foliage (and bark for selected sample trees) were measured, iv) foliage mass and total surface area were determined by destructive measurement, and v) the tree without foliage was photographed in the goniometer to obtain the total (surface) area of woody parts, i.e., stem and branches.

The trees belonged to three species: Scots pine (*Pinus sylvestris* L.), Norway spruce (*Picea abies* (L.) H. Karst), and sessile oak (*Quercus petraea* (Matt.) Liebl.). We refer to them as ‘pine’, ‘spruce’, and ‘oak’. In total, we measured six individual trees per each species. The trees were selected to cover maximal variation in the spherically averaged silhouette to total area ratio (STAR). The trees were brought from a local nursery in Zurich, and were stored in the garden of the university during the campaign (from Aug 20th to Sep 20th in 2018). The outdoor garden was in an open area, and the trees were watered frequently. The condition of the trees was monitored and only trees with no visible symptoms of water stress or disease were selected for measurement. Usually one tree, in some cases two, per day was measured. Multiangular spectral measurements were performed within 1–1.5 h, and leaf and needle spectral measurements within approximately 6 h from the start of the measurements.

Section 2.2 explains the measurements in chronological order, and Section 2.3 explains the data processing in the processing order. Section 2.3 also provides the theory and equations for the data processing. Thus, it augments the data description in Section 1. In addition, a Python code that is provided together with the data further clarifies the processing steps through examples.

### 2.2 Data collection

#### 2.2.1 Multiangular spectra of trees

**Fig. 1:**
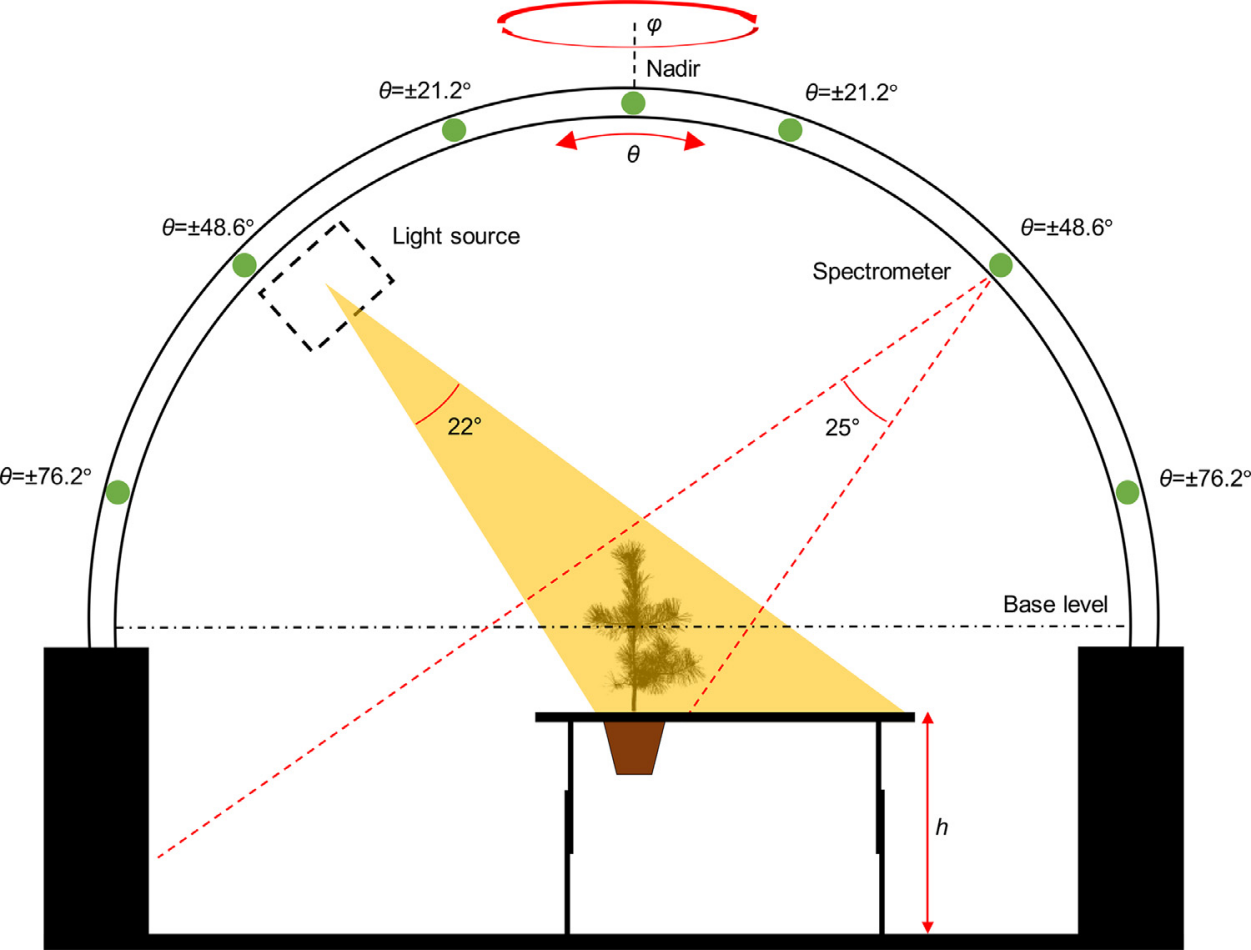
Side-view of the LAGOS goniometer and the measurement setup with the light source and spectrometer. View zenith angles (*θ*) are denoted with green marks on the goniometer's arc. The tree was always exactly in the center of the goniometer, and the vertical mid-point of the tree crown was at the base level, i.e., the light beam and sensor's field-of-view were pointing exactly to the center of the tree crown. Symbol *h* indicates the height of the frame that holds the black background canvas and that was adjusted depending on tree height.

The measurement routine started with measurements of multiangular spectra of the tree in the goniometer. We used the LAGOS (Laboratory goniometer system) goniometer [3,4], which is a large goniometer (radius of 2 m) capable of measuring in all view angles over the hemisphere, excluding zenith angles larger than ∼76° (Fig. 1). To illuminate the trees, we used a 1000 W brightness stabilized tungsten halogen lamp that generated a conical light beam (opening angle of approx. 22°) using a Köhler illuminator with aspherical reflector and a condenser. The lamp was pointing at the tree from 1.75 m distance at zenith angle of 40°, and it illuminated the tree completely (Fig. 1). We used an ASD FieldSpec 3 non-imaging spectrometer (serial number 16006), which measured in the wavelength range of 350–2500 nm, and outputted spectra at 1 nm intervals. All measurements were performed in digital numbers (DN) and converted into physical quantities in post-processing. The spectrometer's detector unit, i.e., a bare fiber-optic bundle with nominal field-of-view (FOV) of 25°, was pointing at the center of the goniometer from 1.94 m distance (Fig. 1). The spectrometer has three separate detectors: visible-near-infrared (VNIR, 350–1000 nm), shortwave-infrared 1 (SWIR1, 1001–1800 nm), and shortwave-infrared 2 (SWIR2, 1800–2500 nm), which have slightly different FOVs, because they view the target through separate optical fibers in the bundle.

**Fig. 2:**
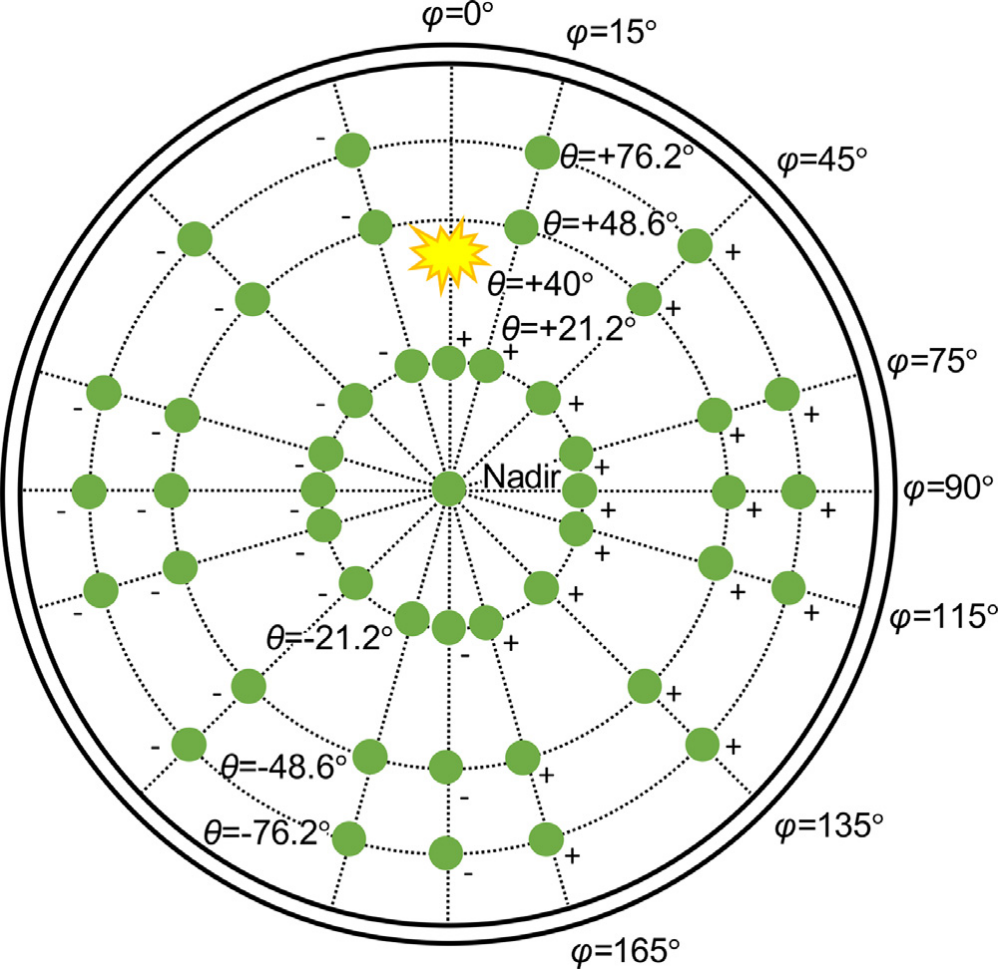
Top-view of the goniometer showing the angle notation used in the data collection. There were eight view azimuth angles (*φ*), and seven view zenith angles (*θ*) per azimuth, except in the principal plane (*φ* = 0°), where view zenith angles behind the lamp could not be measured.

The measurements were performed in eight view azimuth angles (*φ*), and in seven view zenith angles (*θ*) per azimuth (Fig. 2). The view azimuth angles included the principal (*φ* = 0°) and cross-plane (*φ* = 90°), and in addition, six azimuth angles at 15°, 45°, 75°, 115°, 135°, and 165°. The principal and cross-plane were included because they are interesting for interpretation of remote sensing data, and the latter six view azimuth angles were important to obtain a systematic sampling over the hemisphere. The view zenith angles were −76.2°, −48.6°, −21.2°, ±0°, +21.2°, +48.6°, and +76.2°. They correspond to the nodes of Gauss-Legendre integration so that cos*θ* are the Gauss-Legendre weights. The only exception was the principal plane, in which two view zenith angles behind the lamp (*θ =* [+48.6°, +76.2°]) could not be measured because the lamp obstructed the FOV. In total, there were thus 47 different view angles (Fig. 2). Because the measurement in nadir (*φ* = 0°, *θ =* 0°) was performed separately for each azimuth angle, there were seven repetitions of nadir measurement, which resulted in total of 54 measurements per tree. The integration time was 2.18 s for each individual spectrum, and 10 individual spectra were averaged into one measurement. Before and after the measurements of a tree, a white reference was measured in nadir. The white reference was a calibrated Zenith Lite® panel with dimensions of 20 × 20 cm and nominal reflectance of 95%. It was placed in the center of the goniometer, using a tripod. Three white reference measurements were taken both before and after the tree measurements. All white reference measurements per tree were averaged into one value in the data processing.

We placed a spectrally black (Sunbrella® Solid VV M100) acrylic canvas attached to a wooden frame (1.3 m × 1.5 m) below the tree (or white reference), so that the fraction of illumination beam that was not intercepted by the tree (or white reference) was captured by the canvas (Fig. 1). This ensured a well-controlled and predictable background signal (stray light), which could be later removed in the data processing. The canvas had directional-hemispherical reflectance factor (DHRF) of 0.013–0.02 (measured with an integrating sphere). The height of the wooden frame, and thus the canvas, could be adjusted (Fig. 1). We used four pre-defined heights: 0.6 m, 0.65 m, 0.69 m, and 0.74 m. The height of the canvas was selected based on the tree height so that the pot of the tree was below the canvas, while the entire tree crown was above the canvas and fully illuminated by the light beam (Fig. 1). This setup ensured that the signal recorded by the spectrometer was composed of the signal from the tree, and additionally stray light, which mainly originated from the canvas. The amount of stray light depended on the directional scattering characteristics of the canvas, and on the illuminated area seen by the spectrometer (Fig. 1). The stray light was measured once for each view angle and for each frame height. The stray light was ratioed to the white reference measured in nadir view. We call this ratio as ‘stray light fraction’. An estimate of stray light for each tree could then be calculated based on the white reference measurement and stray light fraction.

#### 2.2.2 Multiangular silhouette photographs of trees

After the spectral measurements, red-green-blue (RGB) photographs of the tree were taken in the same 47 view angles as used for the spectral measurements. An additional photograph was taken in the direction of the illumination, while the lamp was moved away to allow the camera to see the tree. The photographs in the view angles were used for calculating STAR, and the photograph in the direction of illumination was used for normalizing the measured spectra to the amount of radiation intercepted by the tree. The photography was performed with a Nikon D5000 camera with an adjustable lens that had focal length set at 45 mm. The camera was attached to the goniometer next to the detector unit of the spectrometer: the view zenith angles of the camera and spectrometer differed 3°, because the physical dimensions of the camera did not allow it to be placed exactly at the same position with the spectrometer. During the photography, the tree was illuminated from both sides with two LED lamps (2 × 10 W each), and a white canvas was placed on the background. This enabled the silhouette of the tree to be separated from the background in data processing. The camera was remotely triggered, and the f-number was *f/8*. To avoid saturation of the photos, exposure time was manually set for each azimuth by underexposing the white background by 0.7 exposure stops, using the built-in light meter of the camera. Photographs taken with −0.7 and +0.7 exposure stops from this default level were always taken also. These additional photographs were only used in evaluating the effect of exposure time on the results [2], and are not included in the data described in Table 1.

#### 2.2.3 Leaf, needle, and bark spectra

Leaf and needle spectra were measured for three samples per tree. A sample refers to a leaf or a set of up to approx. 20 needles (depending on needle dimensions). Leaves and needles were randomly picked and detached from the tree. Only healthy green leaves and needles were measured. For conifers, all needle age classes were mixed randomly. Measurements of spectral directional-hemispherical reflectance (DHRF) and transmittance (DHTF) factors were performed with ASD FieldSpec 3 non-imaging spectrometer (serial number 16007) attached to an ASD RTS-3ZC integrating sphere with a halogen light source. Special sample holders (‘needle carriers’, see Fig. 1 in [5]) were used to attach the needle samples to the integrating sphere. The needle carriers were used also when measuring leaves, to ensure a comparable measurement. The needles were placed in the carrier side-by-side, so that between-needle distance equalled 0.5–1 × needle width. Thus, the number of measured needles depended on needle dimensions. Spruce needles were placed in two rows because they were shorter than the diameter of the sample port in the integrating sphere. The measurement protocol followed the protocol described in [5], except that there the needle carriers were 0.3 mm thick and here 0.8 mm. The protocol for both reflectance and transmittance included measurement of white reference, and measurements of both sides of the sample. The sides were adaxial (‘upper’) and abaxial (‘lower’). Spruce needles were symmetric and no adaxial and abaxial sides could be distinguished. However, both sides of the sample were measured also for spruce needles to ensure equal number of observations for each species. The measurement of reflectance included also a measurement of stray light. All spectral measurements were performed in digital numbers and converted to DHRF and DHTF in the data processing. Integration time in the measurements was 1.09 s for each individual spectrum, and 10 individual spectra were averaged into one measurement. To determinate the gap fractions in the needle samples, we scanned the carriers with needles in them, using Epson Perfection V550 digital film scanner (8-bit grayscale images in 800 dpi resolution).

For one sample tree per species, bark reflectance spectra were measured utilizing the same protocol as described above. Three to five bark samples per tree were peeled off from the tree stem from different heights, and placed in the needle carrier for the measurements. The bark outer surface was then measured for DHRF. Note that one of the bark sample trees (an oak) was taken outside of the set of 18 trees measured in the goniometer, and thus the total number of trees in our dataset is 19 (Table 1). No other measurements were conducted for this one oak tree.

#### 2.2.4 Leaf and needle mass and area

Leaf and needle fresh mass were measured by picking all leaves or needles from the tree and by weighing them with a scale with 1/10,000 g precision. Weighing was performed within 20 min from the picking to avoid excess loss of water. For the purpose of fresh mass to surface area conversion, projection area of a subset of leaves or needles (1 g and 10 g, i.e., approximately 150 needles for pine and spruce, 5 g for oak) from each tree was measured by scanning them in the Epson Perfection V550 scanner (8-bit grayscale images in 800 dpi resolution). Further, another smaller subset (10 needles) was picked from each pine and spruce tree, and measured for needle length and two widths, using a digital calliper with 1/100 mm precision. The two width measurements were made in the middle between the tip and base of each needle, corresponding to the breadth and thickness of the almost half-cylinder-shaped cross section of the pine needles, and two transverse dimensions of the diamond-shaped cross section of the spruce needles.

#### 2.2.5 Multiangular silhouette photographs of trees without foliage

Finally, the tree without its foliage was put back in the goniometer, and the silhouette photography of the woody parts was performed, using the same protocol as for the trees with foliage (Section 2.2.2), except for that only three view azimuth angles (15°, 75°, 135°) were used in order to minimize the measurement time. The total number of photographs was therefore 21 (3 × 7), including two repetitions in nadir, i.e., there were 19 unique view angles.

### 2.3 Data processing

#### 2.3.1 Multiangular silhouette areas of trees

The multiangular silhouette photographs (Sections 2.2.2 and 2.2.5) were thresholded to yield black-and-white images by applying an implementation of the Otsu's method [6] in Matlab software to the blue channel of the photographs. The thresholding was preceded by manual drawing of a polygon (in each photograph) that contained only the tree and the white background. The processing chain was the same for both with and without foliage, except that in the photographs without foliage some areas were erroneously detected as tree by the Otsu's method and needed to be removed manually. This was because the contrast between the tree and the background was not always perfect. The resulting black-and-white images were converted into silhouette areas (*S_tree_*, [m^2^]) by multiplying the number of pixels belonging to a tree with the pixel size at the distance of the tree (0.06377 mm^2^). The pixel size was obtained utilizing the camera's intrinsic (focal length and pixel size) and extrinsic (position and orientation) parameters. These parameters were obtained using photographs taken of a black-and-white checkerboard target in each view angle, and processing with Computer Vision Toolbox™ in Matlab. The processing comprised of performing camera calibration utilizing all photographs, and then solving the position and orientation of the camera in each view (and illumination) angle separately.

#### 2.3.2 Processing multiangular spectra of trees

Multiangular spectra (Section 2.2.1) were processed into estimates of directional scattering coefficients (DSC*_tree_*(Ω), [sr-1]). Note that due to the biconical geometry of the measurements (Fig. 1), our processing results in an approximation of true DSC*_tree_*(Ω) that would be observed in an infinitesimally narrow solid angle. The DSC*_tree_*(Ω) gives the probability density of scattered photons (per steradian) in a given view direction Ω, or, in other words, the fraction of intercepted photons scattered into a unit solid angle around Ω. Multiplication of DSC*_tree_*(Ω) by *π* gives the ratio of signal measured from the tree to that measured in nadir view from an ideal (non-absorbing) Lambertian surface of same surface area. This is conceptually similar to the bidirectional reflectance factor (BRF) commonly used for quantifying the scattering by surfaces in remote sensing [7]. Alternatively, multiplication of DSC*_tree_*(Ω) by 4*π* gives the ratio of signal measured from the tree to that measured from an ideal isotropic scatterer that scatters in all spherical directions. Note that for simplicity, we have omitted the wavelength sign of the spectral quantities in the equations presented in Sections 2.3.2 and 2.3.3.

The computation of DSC*_tree_*(Ω) has been reported in Hovi et al. [2] and in Forsström et al. [1]. For completeness, we provide the basic computation steps also here. For derivation of the measurement equations and estimation of uncertainties in DSC*_tree_*(Ω), see [2]. The equation for DSC*_tree_*(Ω) is

(1) $$\text{DS}C_{tree}\left(\Omega \right)=\frac{DN_{tree}\left(\Omega \right)}{DN_{\text{WR}\_tree}}\times \frac{S_{\text{WR}\_tree}\cos40^{\circ }}{S_{tree}\left(\Omega_{i}\right)}\times \frac{R_{\text{WR}\_tree}\cos0^{\circ }}{\pi }\times \frac{f_{\text{WR}\_tree}}{f_{tree}\left(\Omega \right)},$$

where DN*_tree_*(Ω) and DN_WR_*__tree_* are the measured signals [digital number] from the tree and white reference (white reference was always measured in nadir view), *S_tree_*(Ω*_i_*) is the silhouette area of the tree in the direction of illumination [m^2^], *S*_WR_*__tree_* is the surface area of the white reference panel [m^2^], *R*_WR_*__tree_* [fraction] is the reflectance of the white reference panel, and *f*_WR_*__tree_* and *f_tree_*(Ω) [fraction] are estimates of the ratio of the measured signal to that measured by an isotropic detector, for the white reference and the tree, respectively. To explain shortly, Eq. (1) calculates the ratio of signals from the tree and white reference (first term on the right-hand side), multiplies it with the ratio of the radiation intercepted by the white reference and the tree (second term), multiplies the result with the DSC of the white reference panel at nadir (third term), and finally multiplies the result with a correction factor (fourth term) that takes into account the spectrometer's sensitivity within its FOV (i.e., detectors of the spectrometer had approximately Gaussian point-spread-functions with sensitivity falling off away from the center towards the edges). The derivation of the correction factors *f*_WR_*__tree_*/*f_tree_*(Ω), one for each of the three detectors, has been explained in Section 3.6.2 of [2] and in Section 2.3.2.2 of [1].

Eq. (1) assumes that DN*_tree_*(Ω) and DN_WR_*__tree_* are free of stray light. Stray light fraction in each view angle was known from the measurements of an empty goniometer, i.e., the background canvas in place but without the tree. Thus, the stray light could be computed for each tree based on the white reference measurement. However, the tree (or white reference panel) and its shadow partly covered the illuminated background, and thus obscured a fraction of stray light. For an accurate stray light removal, we used the formulae

(2) $$\begin{array}{ccc} & & DN_{tree}\left(\Omega \right)=DN_{total,tree}\left(\Omega \right)-b_{tree}\left(\Omega \right)DN_{stray}\left(\Omega \right),\\ & & \text{and} \end{array}$$

(3) $$\;\;DN_{\text{WR}\_tree}=DN_{total,\text{WR}\_tree}-b_{\text{WR}\_tree}DN_{stray,\text{WR}\_tree},$$

where DN*_tree_*(Ω) and DN_WR_*__tree_* are the signals from the tree and white reference free from stray light, DN*_total,tree_*(Ω) and DN*_total_*_,WR_*__tree_* are the signals from the tree and white reference that contain stray light, DN*_stray_*(Ω) and DN*_stray_*_,WR_*__tree_* are stray light that would be measured in an empty goniometer (calculated based on the white reference measurements and stray light fraction (DN*_stray_*(Ω)), or measured separately for each tree (DN*_stray_*_,WR_*__tree_*)), and *b*_tree_(Ω) and *b*_WR_*__tree_* are the fractions of stray light not obscured by the tree or white reference panel. Calculations of *b*_tree_(Ω) and *b*_WR_*__tree_* were performed for each of the detectors of the spectrometer separately, using the multiangular silhouette photographs, and additionally photographs taken of the light beam. Using photogrammetric techniques, it was possible to calculate the fraction of illuminated background that the spectrometer's detector could ‘see’ in the presence of the tree. The process has been described and illustrated in Section 3.6.3 of [2] and in Section 2.3.2.3 of [1].

Despite the corrections, there remained jumps between the detectors of the spectrometer. In addition, there was high-frequency noise present close to 350 nm and close to 2500 nm. To remove the noise, the spectra were smoothed with a Savitzky-Golay filter [8]. Finally, the sensor jumps were removed by multiplying the spectra obtained by the SWIR1 and SWIR2 detectors by correction factors, which were obtained by comparing the difference of DSC between SWIR1 (1001 nm) and VNIR (1000 nm), and then by comparing the remaining difference between SWIR2 (1801 nm) and SWIR1 (1800 nm). We provide both original (DSC_*tree,*_*_raw_*), as well as jump-corrected and filtered (DSC*_tree,__filt_*) spectra. The former were used by Forsström et al. [1], and the latter by Hovi et al. [2]. Uncertainty of DSC*_tree_*(Ω) is estimated to be 15–30% in relative terms (see Section 3.6.5 of [2] for details). The uncertainty is the highest in the regions were the signal from the tree is at its lowest and thus the contribution of stray light the highest, i.e., in the blue and red wavelengths, and in the water absorption regions in the shortwave-infrared.

An estimate of the tree's hemispherical reflectance (*R_tree_*), i.e., the fraction of intercepted radiation scattered into hemisphere, can be obtained from the DSC*_tree_*(Ω) values by numerical Gauss-Legendre integration. To ensure systematic distribution of observations over the hemisphere, we did not use principal and cross-planes here. The nadir observations were also dropped out because nadir does not belong to the Gauss-Legendre nodes. Thus, *R_tree_* was calculated as

(4) $$R_{tree}=\frac{2\pi }{12}\sum_{i=1}^{12}\sum_{j=1}^{3}w_{j}\text{DS}C_{tree}\left(\Omega_{ij}\right),$$

where *i* are the view azimuth angles, *j* are the view zenith angles, *w_j_* are the Gauss-Legendre weights for each view zenith angle. Note that here we have separated positive and negative zenith angles into separate azimuths. Thus, there are 12 instead of 6 azimuth angles. Multiplication with 2*π* is required because DSC*_tree_*(Ω) is per one steradian, and hemisphere has 2*π* steradians. The calculations of both DSC*_tree_*(Ω) and *R_tree_* from the raw data are demonstrated in the Python code provided with the data.

#### 2.3.3 Leaf, needle, and bark spectra

The leaf and needle spectral DHRF and DHTF, hereafter called simply as reflectance (*R_leaf_*) and transmittance (*T_leaf_*), were computed from the measurements made in an integrating sphere (Section 2.2.3) as

(5) $$R_{leaf}=\frac{DN_{leaf,R}}{DN_{\text{WR}\_leaf,R}}\frac{1}{1-G_{R}}R_{\text{WR}\_leaf},$$

and

(6) $$T_{leaf}=\frac{DN_{leaf,T}-G_{T}}{DN_{\text{WR}\_leaf,T}}\frac{1}{1-G_{T}}R_{\text{WR}\_leaf},$$

where DN*_leaf,#_* and DN_WR_*__leaf,#_* are the readings taken from the sample and white reference, respectively, *R*_WR_*__leaf_* is the reflectance of the white reference, and *G_R_* and *G_T_* are the gap fractions in the sample. Stray light was subtracted from DN*_leaf,R_* before DN*_leaf,R_* was applied in calculation of *R_leaf_*. For oak leaves, gap fractions were zero since the leaf always filled the sample port. Gap fraction in a needle sample was obtained by applying a threshold to the scanned image of the carrier with needles in it, and weighting the obtained black-and-white image with a ‘light mask’ that models the spatial distribution of the irradiance of the light beam on the sample. The procedure has been described in detail in [5]. The optimal threshold value (202 for pine, 187 for spruce) was selected so that, when the resulting gap fraction was applied in Eq. (6), the mean transmittance (*T_leaf_*) at 410–420 nm matched a ‘target value’. The 410–420 nm region was used since in that region needle transmittance is close to zero with small residual variation depending on the sample, and thus the errors of the estimated gap fraction due to assuming constant transmittance are minimized. The target *T_leaf_* values (0.021 for pine, 0.039 for spruce) were obtained in a separate measurement campaign in 2019, for the same species but grown in Finland. In that campaign, the gap fractions of the needle samples were obtained directly through measurements in the integrating sphere, by painting the illuminated side of the needles black, thus ensuring that the measured transmittance signal was only due to the transmission through the gaps between needles [9]. An accurate estimate of needle transmittance could then be derived from measurements made before painting, because the gap fraction was known. Finally, we applied an empirical bias correction to all processed transmittance spectra (i.e., for both leaves and needles) by adjusting *T_leaf_* downwards by 5.5% (in relative terms). The bias correction was taken from the measurements made against a trusted reference method in [5], and it ensured that leaf and needle albedo (*R_leaf_* + *T_leaf_*) did not exceed unity in any of the measurements. Finally, bark reflectance spectra were also processed using Eq. (5).

#### 2.3.4 Leaf and needle area

Total leaf or needle surface area (TA, [m^2^]) for a tree was obtained by multiplying the fresh mass of leaves or needles [kg] with the projected area to fresh mass (PA/FM, [m^2^ kg^−1^]) and total area to projected area (TA/PA, [m^2^ m^−2^]) ratios. The PA/FM ratio was obtained from the scanned and weighed subset of leaves or needles, and the TA/PA ratio for needles was obtained from the subset that had been scanned and measured for dimensions. In order to calculate projected area from the scanned grayscale images, we applied a constant black-and-white threshold of 200. In order to calculate the total needle surface area from the measurements of needle dimensions, the shape of spruce needles was assumed as parallelepiped (Eq. (9) in [10]), and that of pine needles as semi-fusiform (Eq. (7) in [11]). Because the leaves are flat, the TA/PA ratio for leaves was simply two.

#### 2.3.5 Spherically averaged silhouette to total area ratio (STAR) and woody area

Spherically averaged silhouette area of a tree was computed with Gauss-Legendre integration as

(7) $${\bar{S}}_{tree}=\frac{1}{12}\sum_{i=1}^{12}\sum_{j=1}^{3}w_{j}S_{tree}\left(\Omega_{ij}\right),$$

where *S_tree_*(Ω_ij_) is the silhouette area in direction Ω*_ij_*, and *i* and *j* are the azimuth and zenith angles, respectively. For explanation of the other symbols, see Eq. (4). The STAR with foliage only was calculated as

(8) $$\text{STA}R_{foliage}=\frac{{\bar{S}}_{tree}}{TA_{foliage}},$$

where ${\bar{S}}_{tree}$ is the spherically averaged silhouette area of the tree including both foliage and woody parts, and TA*_foliage_* is the total area of foliage in the tree. We also calculated the total area of woody parts for each tree, utilizing the spherically averaged silhouette area without foliage (${\bar{S}}_{tree,wood}$) and assuming that the total woody area (TA*_wood_*) equals four times the spherically averaged silhouette area, which is true for any convex body. It was assumed that there is no self-shadowing, because the branches in the trees were sparse. Finally, STAR with woody parts included (STAR*_all_*) was obtained by applying Eq. (8) but now including both woody parts and foliage in total area (i.e., replacing TA*_foliage_* with TA*_all_=* TA*_foliage_*+TA*_wood_*).

## CRediT Author Statement

**Aarne Hovi:** Conceptualization, Methodology, Investigation, Data curation, Writing - Original Draft; **Petri Forsström:** Methodology, Investigation, Visualization, Writing - review & editing; **Giulia Ghielmetti:** Resources, Writing - review & editing; **Michael E. Schaepman:** Conceptualization, Methodology, Resources, Funding acquisition, Writing - review & editing; **Miina Rautiainen:** Conceptualization, Supervision, Funding acquisition, Project administration, Writing - Review & Editing.

## Declaration of Competing Interest

The authors declare that they have no known competing financial interests or personal relationships which have or could be perceived to have influenced the work reported in this article.
