# Corrigendum to ‘A dataset composed of multiangular spectral libraries and auxiliary data at tree, leaf, needle, and bark level for three common European tree species’ [Data in Brief 35 (2021) 106820]

**DOI:** 10.1016/j.dib.2021.107236

**Published:** 2021-06-18

**Authors:** Aarne Hovi, Petri R. Forsström, Giulia Ghielmetti, Michael E. Schaepman, Miina Rautiainen

**Affiliations:** a Department of Built Environment, Aalto University, School of Engineering, P.O. Box 14100, FI-00076 Aalto, Finland; b Department of Geography, Remote Sensing Laboratories, University of Zurich, Winterthurerstrasse 190, CH-8057 Zurich, Switzerland; c Department of Electronics and Nanoengineering, Aalto University, School of Electrical Engineering, P.O. Box 15500, FI-00076 Aalto, Finland

The authors regret that Eq. 6 in the article is incorrect. The term *G_T_* should be subtracted from the quotient of DN*_leaf,T_*/DN*_WR_leaf,T_* (not from DN*_leaf,T_* in the numerator). The correct equation is:

(6) $$T_{leaf}=\left(\frac{DN_{leaf,T}}{DN_{\text{WR}\_leaf,T}}-G{}_{T}\right)\frac{1}{1-G_{T}}R_{\text{WR}\_leaf}$$

The error was only in the article text and therefore did not influence the data published. The authors would like to apologise for any inconvenience caused.

